# Biohybrid magnetic carrier robots for targeted bacteria release in ulcerative colitis

**DOI:** 10.1016/j.mtbio.2026.103671

**Published:** 2026-09-16

**Authors:** Zhuoyue Wang, Li Ding, Dalin Zhang, Sarthak Misra, Venkatasubramanian Kalpathy Venkiteswaran

**Affiliations:** a Department of Biomaterials and Biomedical Technology, University of Groningen and University Medical Centre Groningen, Groningen, 9713 AV, the Netherlands; b Department of Biomechanical Engineering, University of Twente, Enschede, 7500 AE, the Netherlands; c School of Life Sciences and Agriculture-Forestry, Southwest University of Science and Technology, Mianyang, 621010, China

**Keywords:** Biohybrid robots, Gut microbiota, Hydrogels, Magnetic soft robots, Screen-printing, Ulcerative colitis

## Abstract

Ulcerative colitis (UC) is a chronic inflammatory bowel disease characterized by persistent inflammation and ulcers in the colon and rectum. Notably, UC is commonly associated with microbial dysbiosis while bacterial therapies often fail to achieve durable, site-specific colonization. Here, we develop a biohybrid magnetic carrier robot (MCR) platform that delivers bacteria along programmed trajectories and enables prolonged retention at target sites. The MCR is structured with a thin layer of hard-magnetic soft material that responds sensitively to magnetic fields, and a functional layer of bacteria-loaded hydrogel that preserves bacterial viability and release probiotics at specific pH values. This multi-material system is created via a multi-layer screen-printing technique, which allows precise patterning of functional materials at the submillimeter scale and holds promise for scalable production. Precise wireless navigation is validated in simulated environments using a robotic-arm-assisted magnetic driving system. In vivo studies in mice show that MCR-assisted treatment accelerates clinical recovery, significantly increases gut microbial diversity, and mitigates colon damage. In a DSS-induced acute colitis mouse model, the biohybrid magnetic carrier robots improved body-weight recovery, disease activity, colon length, and histological morphology relative to the disease model and material-only controls, while several outcomes also improved relative to free bacteria-loaded hydrogel. Fecal 16S rRNA gene sequencing further showed increased microbial richness and diversity and a shift toward a more control group community configuration after MCR based bacterial treatment. This controllable delivery strategy provides a modular and versatile platform for bacterial therapies in UC, expanding its translational potential toward the treatment of intestinal diseases and the modulation of gut health.

## Highlights

• The biohybrid robotic system enables magnetically guided, site-specific probiotic delivery in the intestine.
• Multi-layer screen-printing allows submillimeter-scale patterning of biohybrid materials and supports scalable manufacturing.
• Programmed magnetic navigation supports precise trajectory control and prolonged retention at target sites.
• In vivo results demonstrate improved targeting and therapeutic efficacy in a mouse colitis model.

## Graphical abstract

**Figure:**
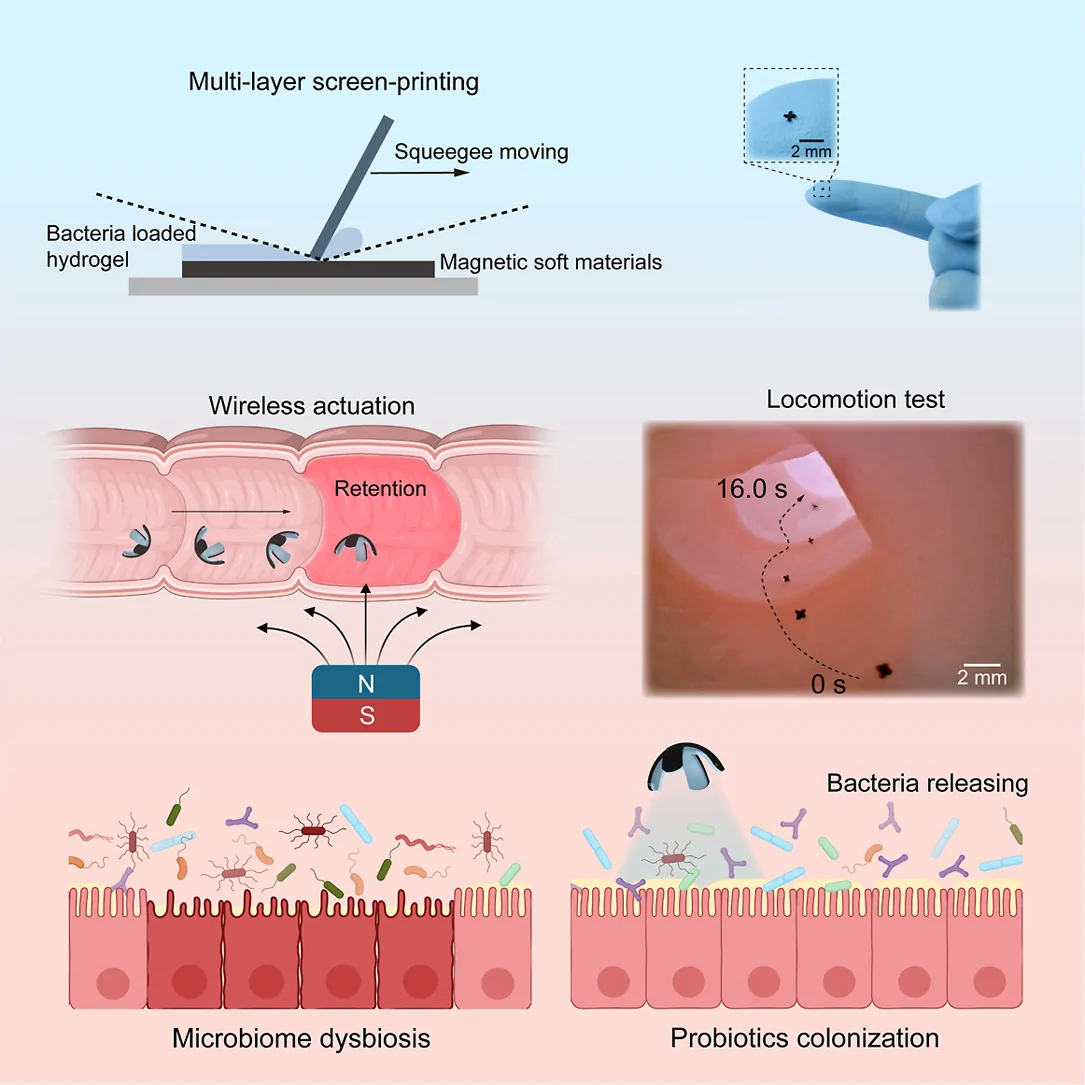

## 1 Introduction

Ulcerative colitis (UC) is a lifelong inflammatory bowel disease (IBD) that affects the rectum and colon, with common symptoms including rectal bleeding in more than 90 % of patients [1,2]. Though the pathogenesis of ulcerative colitis remains incompletely understood, growing evidence indicates that ulcerative colitis is associated with impaired intestinal mucosal barrier function and intestinal microecology dysbiosis [[3], [4], [5], [6]]. Current treatments and maintenance for ulcerative colitis mainly rely on 5-aminosalicylic acid (5-ASA), immunosuppressants (thiopurines), steroids, and antibiotics. These medications can lead to a range of systemic side effects with prolonged use, including nephrotoxicity, hepatotoxicity, and gut dysbiosis [[7], [8], [9]].

Microbiome-based therapies such as fecal microbiota transplantation (FMT) have shown clinical promise in IBD [[10], [11], [12]], including improving symptoms and achieving remission in a subset of patients, but outcomes remain difficult to control. Treatment response varies widely, as strain engraftment and downstream community shifts depend strongly on both donor composition and recipient factors and are not reliably predictable. In addition, safety concerns such as pathogen transmission necessitate stringent donor screening and standardized manufacturing and handling procedures. Engineered probiotics in combination with targeted delivery can help address these limitations by improving consistency, safety, and localization of therapeutic effects. Probiotics can promote microbiota balance and support mucosal barrier function, and probiotic-based approaches have been explored for a range of conditions including inflammatory disorders, rheumatic disease, cancer-associated complications, hypertension, and diabetes [[13], [14], [15], [16]]. In particular, commensal bacteria from the families *Enterobacteriaceae*, *Lactobacillaceae*, and *Bifidobacteriaceae* have been evaluated as therapeutic or adjunctive agents in IBD and shown to modulate cytokine production (e.g., IL-10), IgA responses, and T_reg_-cell-mediated immune regulation [[17], [18], [19], [20], [21], [22], [23], [24]]. Although commonly used as a clinical route of administration, orally administered probiotic or live bio-therapeutic formulations often lose viability during gastric transit and bile exposure. Rapid transit, immune clearance, and competition with resident microbiota further limit colonization and therapeutic durability, which can necessitate frequent dosing or protective formulations [25].

To improve intestinal retention and localization of delivered probiotics, a wide range of delivery strategies has been explored, including utilizing of stimuli-responsive hydrogels, gas-generating systems, and biohybrid “living” constructs. Hydrogel matrices can prolong intestinal retention and protect payloads during transit, exemplified by an inulin-based hydrogel system with long retention for colitis therapy and related complications [26]. Materials that directly augment bacterial function further enhance durability after dosing, including ROS-scavenging adhesive probiotic “backpacks” and adhesive “living glue” coatings that promote gut colonization and therapeutic efficacy in colitis models [27,28]. Multicellular self-organized probiotic assemblies provide an additional route to improve acid and bile tolerance and markedly increase intestinal colonization [29]. However, precise control over the spatiotemporal trajectory of probiotics remains limited in current therapeutic interventions. Without the ability to actively steer and program the delivery path, probiotics are prone to off-target exposure and dilution before reaching the infected target. Therefore, developing a platform with programmable locomotion is essential to transition from uncontrolled delivery to a site-specific therapeutic strategy.

**Fig. 1:**
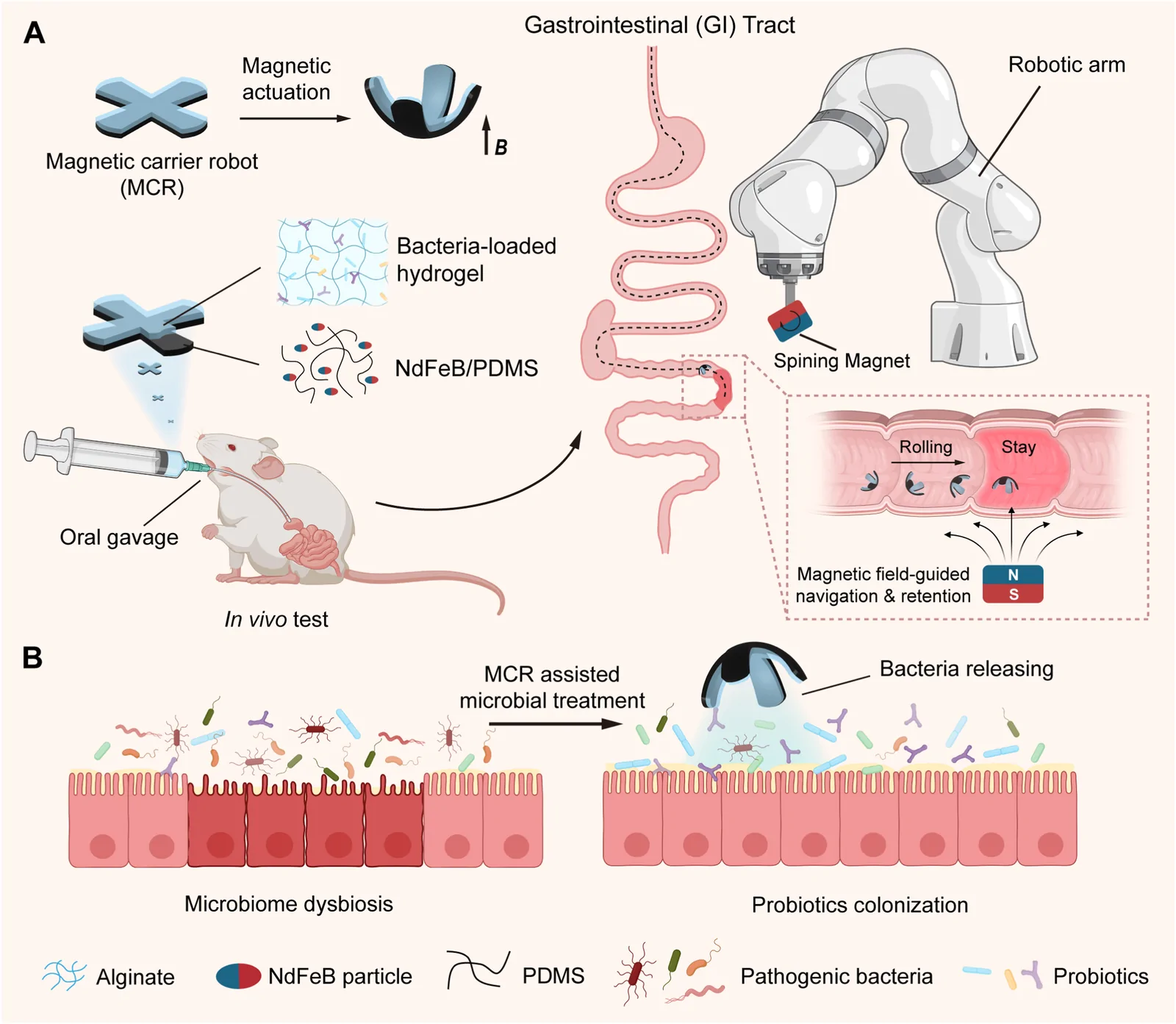
Schematic of magnetic carrier robots (MCRs) for localized probiotic delivery in the gastrointestinal tract. (A) Design and operating concept of MCRs. Each MCR comprises a magnetic elastomer layer of neodymium-iron-boron (NdFeB) particles embedded in polydimethylsiloxane (PDMS) and a probiotic-loaded alginate hydrogel layer. After oral gavage, a spinning permanent magnet on a robotic arm is programmed to generate a rotational magnetic field B) that guides the MCRs for navigation and targeted retention at specific sites. (B) Therapeutic mechanism schematic. MCR-assisted microbial therapy delivers concentrated probiotics directly to the diseased epithelium, enhancing local colonization and helping restore microbiome balance compared with dysbiosis in untreated ulcerative colitis (UC).

Magnetic actuation provides a path toward deterministic delivery. For example, magnetically driven capsules and magnetic soft robots can be navigated and anchored in the GI tract and can support on-demand functions such as tissue attachment and triggered release [[30], [31], [32], [33], [34], [35]]. Here, we propose a site-specific probiotic delivery strategy using magnetic carrier robots (MCRs) with a biohybrid structure that enables wireless control and bacteria release (Fig. 1A). In this study, a bi-layer architecture is designed for the MCRs. The outer layer is a polydimethylsiloxane (PDMS) polymer embedded with neodymium–iron–boron (NdFeB) particles to enable wireless magnetic actuation and compliant contact, and the inner layer is a probiotic-loaded hydrogel that protects bacteria from pH stress and supports localized release after prolonged retention at the target site. For precise positioning of the MCRs in GI tract, a wireless actuation platform based on a robotic arm is developed. By varying the magnet's position and rotation, the external field generates magnetic torque and force on the NdFeB/PDMS layer, thereby enabling guided rolling locomotion. This allows the MCRs to navigate through tortuous lumens and be retained at specific sites on demand. A focused local magnetic field further promotes magnetic trapping and prolonged retention at the target region for site-selective microbial delivery. Positioning accuracy and retention are evaluated in multiple colon-mimicking test tracks and a colon phantom, demonstrating flexible, controllable locomotion and stable on-demand localization.

Additionally, therapeutic efficacy is assessed in a dextran sulfate sodium (DSS)-induced colitis model in live mice following magnet-localized administration. The *in vivo* validation is achieved by magnet-localized oral delivery of MCRs, which accumulate at inflamed colonic regions under external magnetic control and deliver a high local probiotic dose to support engraftment and retention (Fig. 1B). Colitis severity is reduced, as evidenced by improved disease activity index and body-weight recovery, preservation of colon length, ameliorated histopathology (H&E staining), and microbiome modulation quantified by 16S rRNA sequencing. This work establishes a programmable delivery strategy for living substances enabled by magnetic field-controlled robots, showing precise localizing and prolonged intestinal retention, and provides a generalizable framework for future gastrointestinal robotics and targeted microbiome-based therapies.

## 2 Results and discussion

### 2.1 Fabrication and characterization of MCRs

#### 2.1.1 Fabrication of MCRs based on multi-layer screen-printing

**Fig. 2:**
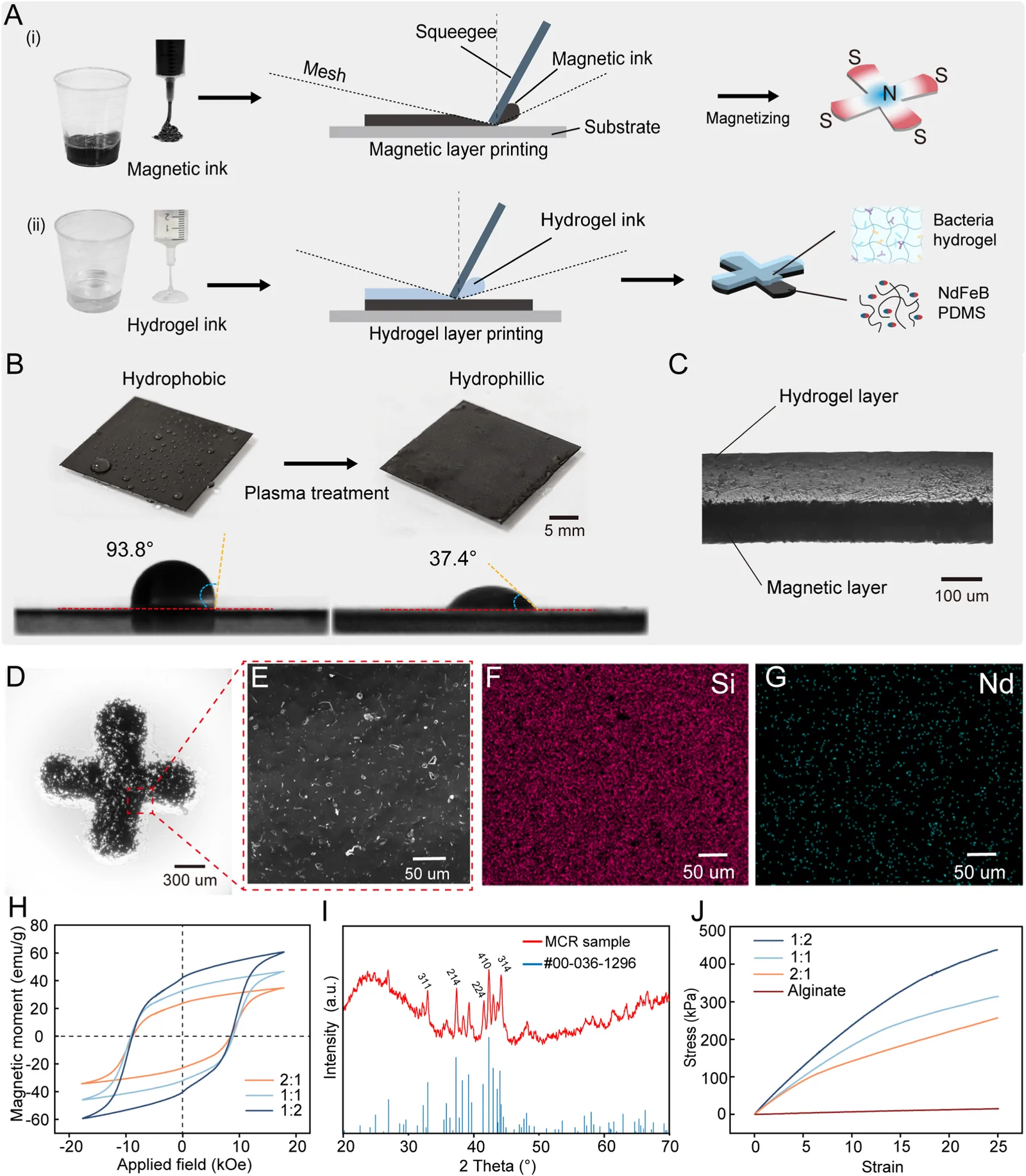
Fabrication and characterization of MCRs. (A) Schematic of the two-step screen-printing process in which a NdFeB/PDMS magnetic ink is first patterned through a mesh onto the substrate, cured, and magnetized, followed by printing of the bacteria-loaded alginate hydrogel ink on top of the magnetic layer. (B) Photographs and contact-angle measurements of the NdFeB/PDMS surface before and after oxygen plasma treatment, showing a transition from hydrophobic (93.8°) to hydrophilic (37.4°), and (C) optical cross-sectional image of the resulting alginate-magnetic bi-layer structure. (D) Optical micrograph of a cross-shaped MCR (scale bar: 300 μm). (E) Scanning electron microscopy (SEM) image of the magnetic layer, and corresponding EDS elemental maps of (F) Si and (G) Nd, showing homogeneous dispersion of NdFeB particles in the PDMS matrix. (H) Magnetic hysteresis loops of NdFeB/PDMS composites with different NdFeB: PDMS mass ratios (1:2, 1:1, and 2:1), demonstrating tunable remanent magnetization and saturation magnetization. (I) X-ray diffraction XRD pattern of the MCR sample compared with the Nd_2_Fe_14_B reference pattern (JCPDS 00-036-1296). (J) Uniaxial tensile stress-strain curves for NdFeB/PDMS composites with different particle loadings and for alginate hydrogel.

MCRs are fabricated using a two-step screen-printing process developed in previous study that sequentially patterns a magnetic elastomer layer and a bacteria-loaded alginate hydrogel (Fig. 2A) [36]. First, using a screen-printing setup illustrated in Fig. S1, magnetized NdFeB particles (∼50 μm) are dispersed in liquid PDMS to obtain homogeneous magnetic ink. This ink is flooded across a stenciled nylon mesh and driven through the open areas onto the substrate by a squeegee pass, forming the magnetic layer. For optimized printability, rheological properties of magnetic ink are modified by adjusting the mass ratio of magnetized NdFeB particles while keeping balance with sufficient magnetic responses (Fig. S2). The printed films are cured for 10 min at 80 °C and then magnetically-programmed as illustrated in Fig. 2A. Because the cured NdFeB/PDMS surface is hydrophobic (water contact angle 93.8°), the robots were subjected to oxygen plasma treatment, which reduced the contact angle to 37.4° and thereby improved wetting and adhesion of the aqueous hydrogel ink (Fig. 2B).

For the hydrogel layer, a freeze-dried cocktail of probiotic strains is resuspended in 10 wt % sodium alginate to obtain viscous, shear-thinning bacterial ink suitable for screen printing. The hydrogel ink is loaded onto a second mesh carrying the desired stencil and, after a flooding step, is printed directly onto the plasma-treated magnetic layer using a controlled squeegee stroke. The deposited ink spread and self-leveled to form a continuous coating with a thickness of approximately 100 μm, as confirmed by cross-sectional imaging (Fig. 2C). Immediately after printing, the patterned hydrogel is sprayed with 0.1 M CaCl_2_ mist, which rapidly cross linked the alginate network and immobilized the bacteria within a mechanically stable layer on top of the magnetic substrate.

#### 2.1.2 Characterization of magnetic layer

After fabrication, the morphology and composition of MCRs are first examined via an optical micrograph (Fig. 2D). A representative cross-shaped robot is shown, demonstrating that the screen-printing process produces well-defined microscale patterns. Dimensional reproducibility is essential for maintaining consistent magnetic actuation, locomotion, and bacterial loading during manufacturing scale-up. OpenCV-based analysis of 1280 MCRs from five independent batches showed that more than 95 % of the printed structures exhibited length and width deviations of no more than 15 % from the median, demonstrating robust process consistency under the current screen-printing conditions (Fig. S3).

Scanning electron microscopy (SEM) image with elemental further reveals a continuous NdFeB/PDMS elastomer layer in which the magnetic particles are finely dispersed within the polymer matrix, with no visible aggregation at the microscale (Fig. 2E and Fig. S4). Elemental mapping by energy-dispersive X-ray spectroscopy further confirms this composite structure. The Si map displays a uniform signal that corresponds to the PDMS phase, whereas Nd appeared as densely and evenly distributed spots, indicating homogeneous dispersion of NdFeB particles within the PDMS matrix (Fig. 2F and G).

The magnetic properties of the composite elastomers are characterized for different NdFeB to PDMS mass ratios (1:2, 1:1, and 2:1). The hysteresis loops show typical ferromagnetic behavior with finite coercivity and remanent magnetization for all three mixing ratios (Fig. 2H). Increasing the particle loading results in a monotonic rise in saturation magnetization and remanent magnetization, with the 2:1 composite exhibiting the highest magnetic moment over the entire applied field range. The 1:1 and 1:2 inks display lower magnetization, consistent with dilution of the magnetic particles by the elastomer. These results indicate that the magnetic responses of the MCRs can be tuned by adjusting the NdFeB content, while preserving sufficient coercivity for stable programmed magnetization.

The XRD pattern of the PDMS/NdFeB composite shows the characteristic diffraction peaks of NdFeB (Fig. 2I), which match well with the standard Nd_2_Fe_14_B phase (JCPDS 00-036-1296). The main peaks observed in the experimental pattern correspond to the tetragonal Nd_2_Fe_14_B crystalline structure, indicating that NdFeB powder is the dominant crystalline filler in the PDMS matrix.

The mechanical properties of the magnetic layer are evaluated by uniaxial tensile testing and compared with those of the alginate hydrogel. The stress-strain curves indicate that the load-bearing capability of the NdFeB/PDMS composites increases with particle content within the tested range (Fig. 2J). At 25 % strain, the stresses increased to 257.1 kPa (NdFeB:PDMS = 1:2), 314.3 kPa (NdFeB:PDMS = 1:1), and 438.2 kPa (NdFeB:PDMS = 2:1), while alginate reached 15.24 kPa. These results indicate that the magnetic layer provides the primary stiffness to support programmed magnetization and actuation, while the overlying alginate hydrogel contributes to a compliant, cell-friendly environment for encapsulated probiotics.

The durability of the MCRs is further evaluated after long-term exposure to simulated gastric fluid (SGF) without pepsin under different pH conditions. During the test, no obvious structural damage or degradation is observed, indicating strong acid–base tolerance. This stability highlights the suitability of MCRs for operation in the gastrointestinal tract, where pH varies substantially across different regions and physiological states (Fig. S5).

#### 2.1.3 Characterization of bacteria-loaded hydrogel ink and release test

**Fig. 3:**
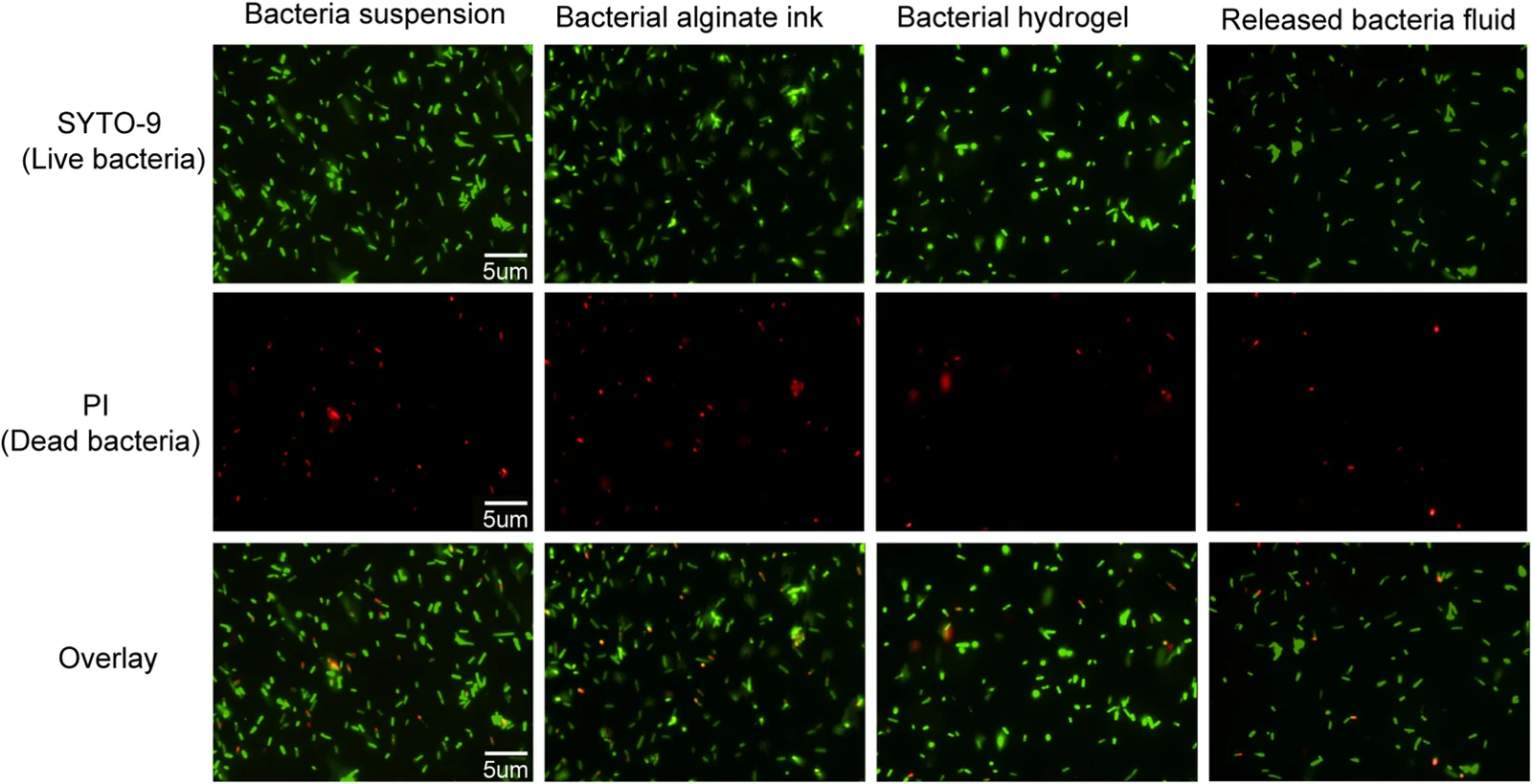
Viability of probiotics during ink formulation, hydrogel crosslinking, and dissolution. Representative fluorescence micrographs of probiotic bacteria stained with SYTO-9 (live, green) and propidium iodide (dead, red) under four conditions: bacteria in PBS (Bacteria + PBS), bacteria dispersed in alginate printing ink (Bacterial alginate ink), bacteria encapsulated in crosslinked alginate hydrogel (Bacterial alginate gel), and bacteria released after dissolution of the hydrogel (Dissolved bacterial alginate gel). Overlay images show that bacterial populations remain predominantly SYTO-9-positive throughout the printing and release process, indicating high viability is preserved in the alginate-based delivery matrix. (For interpretation of the references to colour in this figure legend, the reader is referred to the Web version of this article.)

In addition to demonstrating programmable locomotion and clinically relevant navigation of MCRs, the therapeutic function requires that the MCRs release viable probiotics in a controlled manner after deployment. Therefore, bacterial viability during ink formulation, gelation, and subsequent release is evaluated by live/dead fluorescence staining (Fig. 3 and Fig. S6). In the Bacteria + PBS solution (pH = 6.5), a dense population of rod-shaped cells exhibits bright SYTO 9 fluorescence with only sporadic PI-positive events, consistent with high bacterial viability. Dispersion of bacteria in the 10 wt % alginate ink maintains similar profile, suggesting that exposure to the viscous alginate matrix and mixing imposes minimal additional membrane damage. CaCl_2_ mist supplied Ca^2+^ ions to ionically crosslink sodium alginate, resulting in the formation of a stable bacterial alginate gel. Live/dead staining revealed that the majority of encapsulated bacteria remained SYTO 9-positive, with few PI-positive cells, suggesting that Ca^2+^-mediated gelation and hydrogel confinement had minimal impact on bacterial viability. To emulate bacterial release in the intestine, the gel was subsequently exposed to PBS at pH 7.5, which promoted hydrogel swelling, disintegration, and dissolution. Following dissolution, the released bacterial population remained predominantly green, although a modest increase in red and yellow dual-stained cells was evident relative to the intact gel.

Quantitative spread-plate assay with three independently prepared samples per condition are performed to characterize the viability of loaded bacteria throughout the fabrication process (Fig. S7). We analyzed (i) the reconstituted bacterial suspension, (ii) the bacteria-loaded alginate ink after screen printing, and (iii) the bacteria recovered after CaCl_2_ crosslinking and sodium-citrate de-crosslinking. The measured viable bacterial concentrations were 8.3 × 10^8^ CFU/mL in the reconstituted bacterial suspension, 1.4 × 10^8^ CFU/mL in the bacteria-loaded hydrogel ink after screen printing, and 5.2 × 10^7^ CFU/mL after gelation and release. Although viable bacterial counts decreased during fabrication and gelation, the released suspension still remained 5.2 × 10^7^ CFU/mL, confirming that a viable probiotic population was preserved throughout the preparation and release processes.

### 2.2 Programmable locomotion in ex vivo models

**Fig. 4:**
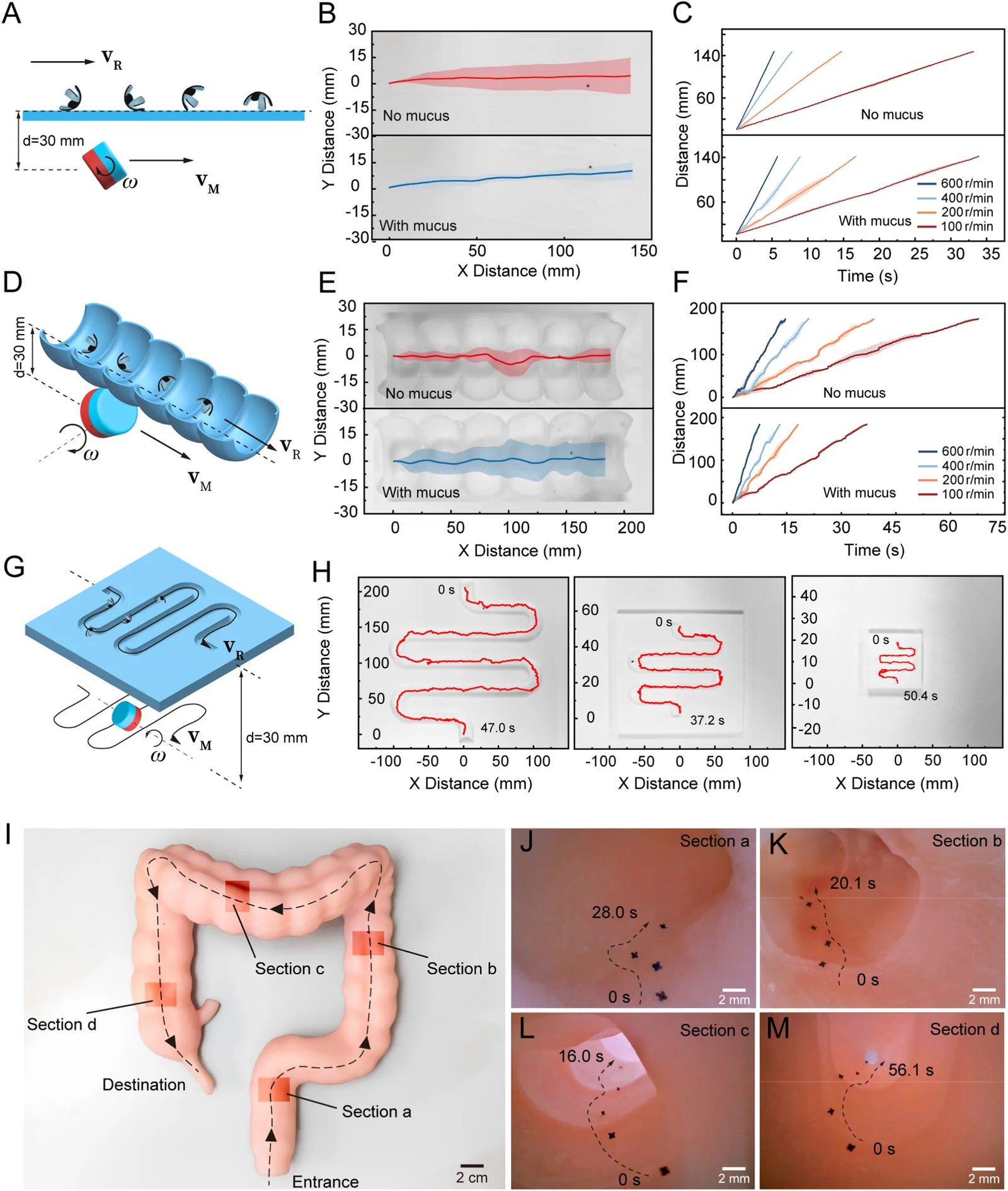
Wireless locomotion and navigation of magnetic carrier robots (MCRs) on test tracks and in a colon phantom. In the schematics, d = 30 mm denotes the center-to-track distance between the disk magnet and the lowest surface of the test tracks. ω is the rotational angular velocity of the magnet. VR indicates the instantaneous velocity direction of the MCR, and VM indicates the instantaneous translational velocity direction of the magnet. (A) Schematic of locomotion of an MCR on a straight track driven by a rotating permanent magnet mounted on a translating robotic arm. (B) Representative snapshot overlaid with the averaged trajectory of an MCR moving along a straight track without mucus and with a mucus layer prepared from simulated gastrointestinal fluid. (C) Travel distance versus actuation frequency on the straight track. (D) Schematic of locomotion in a molded silicone channel with transverse ridges mimicking colonic folds. (E) Representative snapshot overlaid with the averaged trajectory of an MCR moving along a 3D-printed colon-structured track without mucus and with a mucus layer prepared from simulated gastrointestinal fluid. (F) Distance–time plots on the corrugated track at different frequencies. (G) Schematic of navigation in a planar serpentine track guided by coordinated rotation and translation of the external magnet. (H) Representative snapshots overlaid with reconstructed trajectories of an MCR navigating a 3D-printed serpentine track. (I) Schematic of a colon phantom for endoscopy testing, indicating entrance, destination, and four sections (a–d). (J–M) Endoscopic images from sections a–d with overlaid trajectories and time stamps, illustrating wireless guidance of MCRs around bends and through confined regions under clinically relevant visualization, scale bars, 2 mm.

The wireless motion control of the MCRs is characterized by multiple test tracks that mimic key geometric and frictional features of the colon (Fig. 4, Supplementary Movie). The test tracks are designed in CAD and 3D-printed in 3 representative geometries (Fig. S8–S11). The wireless actuation platform based on a robotic arm provides a rotating magnetic field at a prescribed frequency that generates torque and traction on the magnetized MCRs (Fig. S12). MCR trajectories are recorded by an overhead camera and analyzed with an automated tracking pipeline, as illustrated, for instance, by square-path tracking with minor corner rounding and edge deviations (Fig. S13).

On a straight track, the MCR translated along the channel with nearly linear distance–time behavior(Fig. 4A–C). Experiments are performed under two surface conditions: a dry PDMS surface (“no mucus”) and the same surface coated with a thin mucus layer prepared from simulated gastrointestinal fluid (“with mucus”), each tested at external magnet rotation frequencies of 100, 200, 400, and 600 r/min. In both cases, the travel distance increased linearly with time, and higher rotation frequencies produced increased velocities. To evaluate the locomotive performance in more complex geometries, we fabricated a molded silicone track with periodic transverse ridges that mimics human colonic folds (Fig. 4D). The cross-shaped MCR is able to move along the corrugated path in both dry and mucus-covered conditions, as shown by the overlaid trajectories in Fig. 4E. As shown in Fig. 4F, the rolling MCR under 100–600 r/min actuation exhibits a path-dependent near-linear distance–time behavior, with propulsion speed increasing monotonically with increasing actuation frequency. Compared with the dry condition, the mucus-coated surface showed higher average speed at the tested frequencies, suggesting that the mucus modulates traction between MCRs and the colon wall. Controlled navigation in a 3D-printed serpentine planar track is demonstrated (Fig. 4G). By coordinating the rotation and translation of the external magnet along a predefined path, the MCR could be driven from the entrance to the outlet while negotiating multiple bends. The reconstructed trajectories and corresponding elapsed times (Fig. 4H) demonstrate that robots reliably follow the designed path under fully wireless magnetic control, highlighting the feasibility of programmed navigation through simulated intestinal geometries.

To evaluate locomotion in an anatomically relevant environment and simulate rectal administration, MCR navigation is further tested in a colon phantom for endoscopy training that reproduces the gross geometry, curvature, and lumen diameter of the human colon(Fig. 4I). MCRs are introduced through the distal entrance of the phantom and driven toward proximal regions along the prescribed route using an external permanent magnet mounted on a robotic arm, which is rotated and translated outside the phantom following the same actuation scheme used in benchtop tracks. A standard endoscope is advanced through the colonic lumen to visualize the robot in real time, and representative snapshots are collected at four characteristic locations (Sections a–d). In each section, the robot position and trajectory are overlaid within the endoscopic field of view, showing progressive motion over elapsed intervals of 28.0 s (Section a), 20.1 s (Section b), 16.0 s (Section c), and 36.1 s (Section d) from the initial frame at 0 s (Fig. 4J–M). These results demonstrate that MCRs remain trackable under endoscopic imaging and can be magnetically-guided along the folded colonic lumen, around bends, and through locally narrowed regions, supporting the feasibility of integrating wireless magnetic actuation with clinically relevant rectal delivery workflows.

MCRs can be deployed through either oral gavage or rectal enema depending on the target anatomy and intestinal accessibility. Magnetic positioning is therefore adapted to the administration route. For large lumens such as the human colon, rectal placement provides a practical route to position the robots directly within the distal colon, where an external permanent magnet mounted on a robotic arm can assist actuation and guidance. In small animals with narrow colonic dimensions (e.g., mice), device insertion is generally limited to oral gavage for gastrointestinal entry. In this setting, robotic-arm translation offers limited positioning precision because the arm's motion error can be comparable to, or larger than, the organ scale. Accordingly, a permanent magnet is affixed at a predefined external location to enhance local magnetic trapping and prolong intestinal retention at the target site.

Clinical translation of the MCR platform will require adaptation to the larger dimensions and greater working distance of the human colon. Because the magnetic field strength and gradient available for actuation decrease as the distance between the external magnetic source and the MCR increases, human-scale application will require a sufficiently powerful magnetic actuation system combined with real-time imaging and closed-loop position control. Meanwhile, the larger human colonic lumen may permit the use of proportionally larger MCRs, potentially increasing their magnetic moment, imaging visibility, and probiotic-loading capacity while remaining within clinically acceptable dimensional limits. These design advantages should be further evaluated under anatomically and physiologically relevant conditions, including realistic colonic geometry, luminal contents, and intestinal motility. Future translation will also require verified device passage or retrieval, repeated-dose biocompatibility, sterility, reproducible manufacturing, and regulatory assessment of the integrated device-material-probiotic system. Therefore, the present study provides preclinical proof of concept and a foundation for the subsequent development of an image-guided, human-scale MCR delivery platform.

### 2.3 Therapeutic effects of MCRs delivered probiotics against DSS-induced ulcerative colitis

**Fig. 5:**
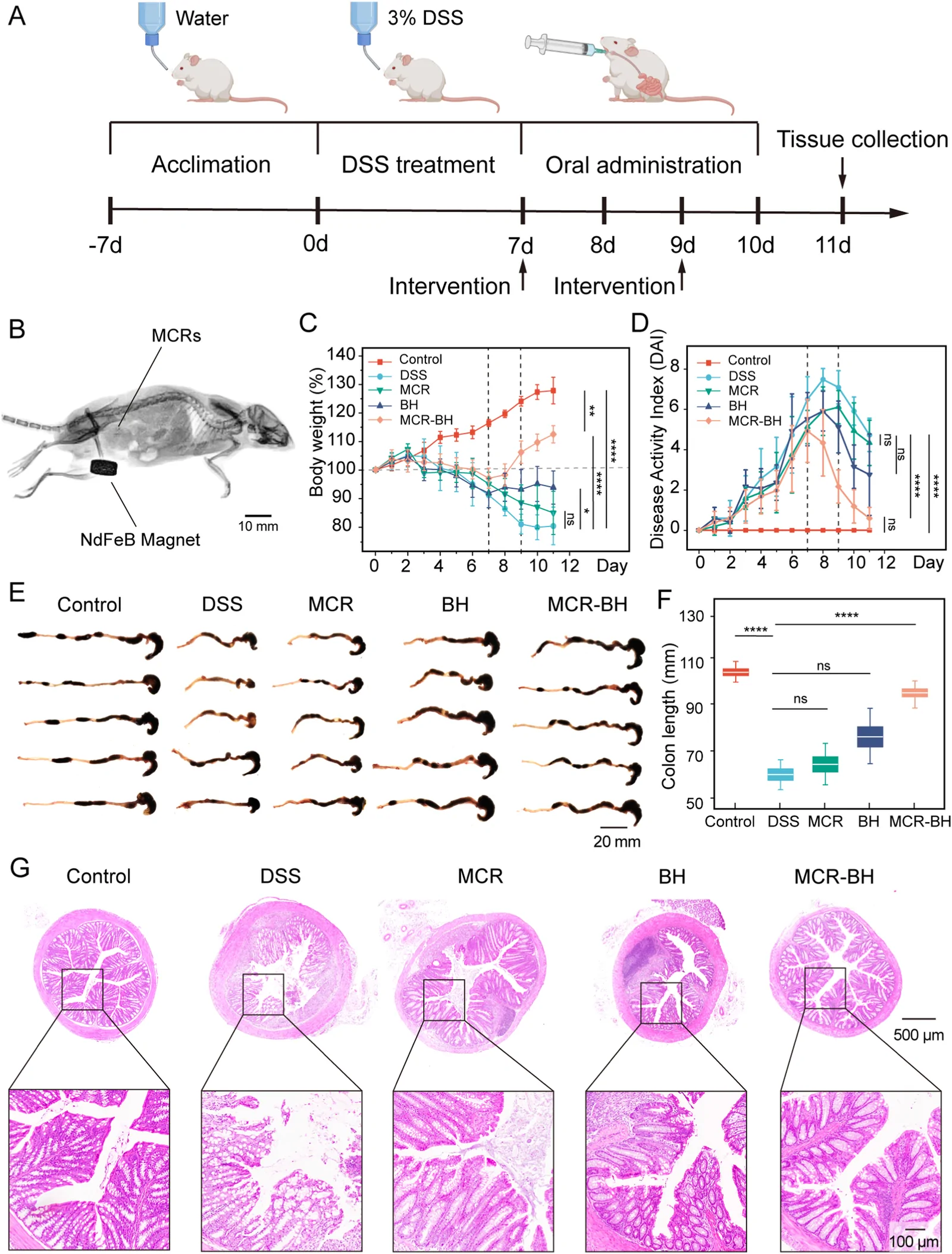
In vivo test of dextran sulfate sodium (DSS)-induced ulcerative colitis by the biohybrid MCRs. (A) Experimental timeline: 7 d acclimation, 3 % DSS in drinking water from day 0-7, oral gavage of blank MCRs, probiotic hydrogel (BH), or probiotic-loaded MCRs (MCR-BH) on days 7 and 9, and tissue collection on day 11. (B) Schematic of *in vivo* magnetic localization, with an external NdFeB magnet positioned over the distal colon. (C) Changes in body weight (percent of baseline) for Control, DSS, MCR, BH, and MCR-BH groups. Significance between groups was determined using a one-way ANOVA followed by Tukey's multiple comparisons test. Data are presented as mean ± SEM (n = 5 per group). Statistical significance is indicated by p values: *p < 0.05, **p < 0.01, ***p < 0.001, and ****p < 0.0001; ns, not significant. (D) Disease activity index (DAI) versus time for the same groups. Significance between groups was determined using a one-way ANOVA followed by Tukey's multiple comparisons test. Data are presented as mean ± SEM (n = 5 per group). Statistical significance is indicated by p values: *p < 0.05, **p < 0.01, ***p < 0.001, and ****p < 0.0001; ns, not significant. (E) Representative macroscopic images of colons harvested on day 11. (F) Quantification of colon length (box-and-whisker plots) showing DSS-induced shortening and its attenuation by BH and, most prominently, by MCR-BH. (G) Representative H\&E-stained distal colon sections, illustrating severe epithelial damage and inflammatory infiltration in DSS and MCR groups, partial improvement with BH, and substantial restoration of mucosal architecture in the MCR-BH group.

To evaluate the therapeutic efficacy of probiotic-loaded magnetic carrier robots, ulcerative colitis is induced in live mice by administering 3 wt % dextran sodium sulfate (DSS) in the drinking water for 7 consecutive days (Fig. 5A). All mice are randomly assigned to five groups: Control, DSS, MCR, BH, and MCR-BH. The Control group received normal drinking water without DSS. The DSS group received DSS to establish colitis without further intervention. The MCR group received DSS followed by oral gavage of blank robots, with an external magnet placed on the abdomen to promote distal-colon localization and retention for 2 days. The BH group received DSS followed by oral gavage of 1 mL probiotic-loaded hydrogel (∼10^8^ CFU). The MCR-BH group received DSS followed by oral gavage of MCRs with 1 mL probiotic-loaded hydrogel, and an abdominal magnet is similarly applied to enhance local trapping and retention for 2 days. MCRs are delivered by oral gavage because precise *in situ* positioning is constrained by the small colonic diameter in mice.

The MCR capturing and retention experiment is further characterized by X-ray and computed tomography (CT) (Fig. 5B and Fig. S14). Therapeutic efficacy is first assessed by tracking body-weight changes across groups throughout the experimental period, as body weight serves as a sensitive indicator of disease onset and progression in DSS-induced colitis. As shown in Fig. 5C, the healthy control group exhibited a steady increase in body weight throughout the study, reaching 126 % of the initial weight by day 11. During DSS induction (day 0-7), body-weight loss is comparable across all DSS-exposed groups. After treatment administration on days 7 and 9, the DSS group continued to decline and reached 80 % of the initial weight by day 10, free BH showed only partial recovery to 93-95 %, and MCR provided minimal benefit with body weight around 85 %, whereas MCR-BH manifests a clear rebound and recovered to 110 % by day 11.

Disease activity index (DAI) trajectories reflected clinical onset, peak severity, and recovery of DSS colitis (Fig. 5D). During DSS induction (day 0-7), the average DAI increased similarly across all DSS-exposed groups from 0 to a peak on day 8. After treatment on days 7 and 9, group differences are evident at the peak disease stage and at the endpoint. On day 8, DAI is 7.6 in DSS, 6.0 in MCR and free BH, and 4.4 in MCR-BH. By day 11, DAI remained 4.8 in DSS and 4.4 in MCR but decreased to 2.8 with free BH and to 0.6 with MCR-BH. The overall decline in DAI after its peak is consistent with symptom alleviation following DSS withdrawal, whereas the differences among groups indicate additional treatment-related effects. Compared with the DSS group, MCR-BH produced a faster and more pronounced clinical recovery, while BH also improved recovery but to a lesser extent than MCR-BH.

Macroscopic assessment of colon morphology further supported these trends (Fig. 5E and F). DSS exposure produced marked colon shortening and wall thickening relative to healthy controls. MCR did not improve this phenotype, whereas free BH provided partial restoration. In contrast, colons from the MCR-BH group appeared longer and less edematous, resembling control tissue. Colon-length quantification yielded 105.9 ± 3.6 mm for Control, 62.0 ± 5.8 mm for DSS, 66.4 ± 7.6 mm for MCR, 78.2 ± 10.0 mm for free BH, and 97.0 ± 4.2 mm for MCR-BH. Histological analysis provided microscopic evidence of mucosal protection (Fig. 5G and Fig. S15). Control slides displayed an intact epithelium, well-organized crypts, and minimal inflammatory infiltration, whereas DSS challenge caused epithelial erosion, crypt loss, ulceration, and dense inflammatory cell infiltration. MCR showed similar lesions, and free BH produced partial improvement with focal crypt regeneration but persistent inflammation. Colons from the MCR-BH group preserved epithelial continuity, exhibited more regular crypt architecture, and showed reduced inflammatory infiltrates, approaching near-normal tissue organization. The improvements in body weight, DAI, colon morphology, and histological integrity indicate that bacteria-loaded MCRs mitigated DSS-induced colitis more effectively than free probiotics or material controls. This conclusion is based on an acute DSS mouse model, and durability and dose calibration should be evaluated in chronic and more clinically relevant settings.

After administration, the free probiotic hydrogel was excreted in the feces within 12 h, whereas MCR-BH remained detectable in the colonic region for up to 48 h under magnetic retention. Compared with the free-hydrogel and material-only MCR controls, MCR-BH exhibited prolonged colonic retention and greater therapeutic improvement in DSS-induced colitis, demonstrating enhanced efficacy of the magnetically retained probiotic delivery system under the tested conditions. Compared with conventional microbiome-based interventions for UC, such as fecal microbiota transplantation and conventional oral probiotic administration, MCR-BH enables the delivery of a defined and selectable probiotic composition, reduces bacterial exposure to gastric acid, minimizes procedural invasiveness, confines probiotic delivery to the targeted colonic region, and prolongs local retention.

In addition to therapeutic efficacy, the potential local and systemic safety risks of MCR administration must be considered before clinical translation. The flexible PDMS matrix may reduce mechanical stress on the intestinal mucosa, while embedding the NdFeB particles within the polymer limits their direct contact with gastrointestinal tissues. In the present mouse study, all administered MCRs were observed to be excreted after removal of the magnet. Moreover, ICP-MS analysis quantified Fe and Nd release in simulated gastrointestinal fluid (Fig. S16), and the Caco-2 cell assay demonstrated good cytocompatibility during the 48-h observation period (Fig. S17). Collectively, these findings provide preliminary evidence of short-term compatibility under the tested conditions. However, the long-term safety, material degradation, and potential immune responses associated with the MCR platform warrant further evaluation in larger animals and humans.

### 2.4 Gut microbiota restoration mechanism of MCRs on UC

**Fig. 6:**
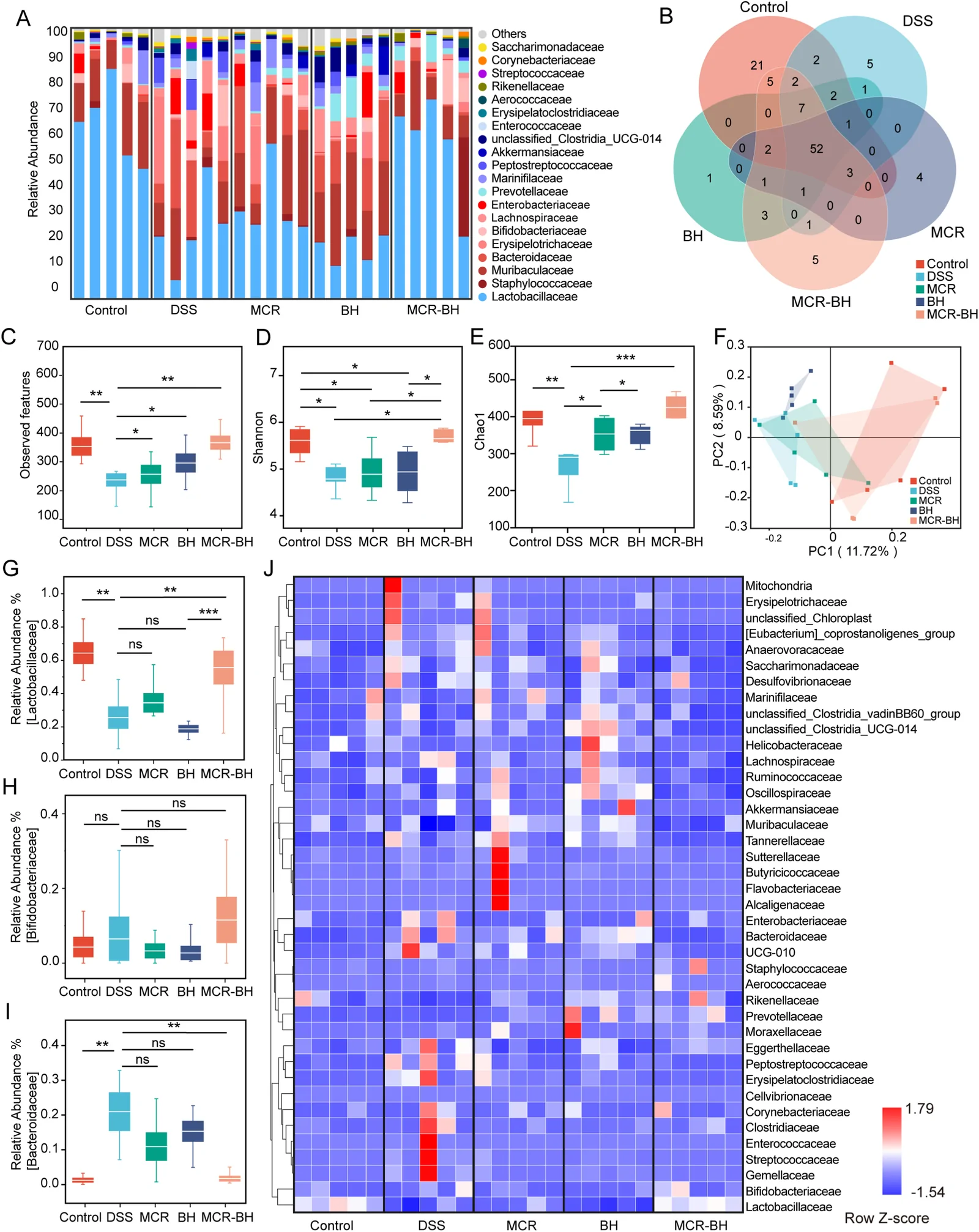
Modulation of gut microbiota by probiotic-loaded magnetic carrier robots (MCR-BH) in DSS-induced colitis. (A) Family-level relative abundance profiles of the fecal microbiota in the Control (red), DSS (light blue), material-only MCR (green), free bacteria-loaded hydrogel (BH, dark blue), and bacteria-loaded MCRs (MCR-BH, pink) groups. (B) Venn diagram showing the shared and group-specific features detected in the Control, DSS, MCR, BH, and MCR-BH groups. Alpha-diversity metrics were used to assess within-sample microbial richness and diversity, including (C) the observed number of features, (D) the Shannon diversity index, and (E) the Chao1 richness estimator. Differences in alpha diversity were evaluated using the Kruskal–Wallis rank-sum test. (F) Beta diversity was calculated using Jaccard distances and visualized by principal coordinates analysis (PCoA), and differences in Jaccard-distance distributions among groups were also evaluated using the Kruskal–Wallis rank-sum test in Fig. S18. (G-I) Relative abundances of selected health-associated and dysbiosis-associated bacterial families, including Lactobacillaceae, Bifidobacteriaceae, and inflammation-associated taxa. Group differences were analyzed by one-way ANOVA followed by Tukey's multiple-comparisons test. Data are presented as the mean ± SEM (n = 5). Statistical significance is denoted as *p < 0.05, **p < 0.01, ***p < 0.001, and ****p < 0.0001; ns, not significant. (J) Heatmap with hierarchical clustering of the dominant bacterial families across all samples, showing that the microbial community profiles of the MCR-BH group clustered more closely with those of the healthy Control group than with those of the DSS and material-only MCR groups. (For interpretation of the references to colour in this figure legend, the reader is referred to the Web version of this article.)

To examine whether probiotic-loaded magnetic carrier robots (MCR-BH) corrected DSS-induced dysbiosis, 16S rRNA gene sequencing is performed on collected feces (Fig. 6). Family-level profiles (Fig. 6A) show that DSS shifted the community away from the healthy pattern, and MCR remained similar to DSS, indicating limited microbiota modulation by the carrier matrix alone. Free ASBH induced only modest remodeling at the family level and largely overlapped with the DSS-like pattern, while MCR-BH more consistently shifted the distribution toward a control-like composition. A Venn diagram of shared and unique feature sequences (Fig. 6B) further summarized overlap across groups. DSS and MCR shared a large set of feature sequences, consistent with a common dysbiosis community structure, whereas MCR-BH showed greater overlap with the healthy control group and a reduced intersection with DSS-associated features, supporting a shift toward a healthier community composition.

Alpha-diversity analysis showed that DSS treatment significantly reduced microbial richness and diversity. The DSS group exhibited significantly fewer observed features than the Control group (Fig. 6C), whereas the MCR, BH, and MCR-BH groups all showed significantly higher numbers of observed features than the DSS group. For the Shannon index, the DSS, MCR, and BH groups had significantly lower values than the Control group (Fig. 6D). In contrast, MCR-BH exhibited a significantly higher Shannon index than the DSS, MCR, and BH groups, with no significant difference from the Control group. Chao1 richness was also significantly lower in the DSS group than in the Control group, while the MCR group showed a significant increase relative to DSS (Fig. 6E). In addition, Chao1 richness was significantly higher in the BH and MCR-BH groups than in the MCR group, with MCR-BH exhibiting the highest median value. Collectively, MCR-BH produced the most consistent improvement across the evaluated richness and diversity metrics.

Beta-diversity analysis revealed marked treatment-associated shifts in the gut microbial community structure. Principal coordinate analysis (PCoA) based on Jaccard distances was used to visualize differences in gut microbial community composition among the treatment groups (Fig. 6F). DSS induced a pronounced deviation of the microbial community from the Control group, consistent with substantial microbiota dysbiosis. Notably, MCR-BH treatment shifted the microbial community toward the Control-associated configuration, indicating a pronounced restoration of the DSS-disrupted microbial structure. Compared with BH treatment, which generated a relatively distinct microbial configuration, the MCR-BH group exhibited a community profile more closely resembling that of the Control group, suggesting a greater restorative effect of the combined treatment on gut microbial dysbiosis. In contrast, the empty MCR carrier did not produce an apparent recovery toward the Control-associated community structure, supporting the notion that the observed microbiota-modulating effect was primarily attributable to the therapeutic intervention rather than the carrier itself. Collectively, these findings indicate that MCR-BH more effectively remodeled the DSS-disrupted gut microbiota toward a healthier community state and provide more effective therapeutic benefit compared with BH alone. As a complementary assessment of beta-diversity dispersion, within-group Jaccard distances were compared across treatments (Fig. S18).

Consistent with broader community-level restoration, family-level analysis showed a significant DSS-associated reduction in *Lactobacillaceae* which is significantly restored by MCR-BH. Importantly, this increase is also significant versus free BH, whereas free BH did not produce a significant improvement over DSS based on the annotated statistics (Fig. 6G). *Bifidobacteriaceae* exhibited modest between-group differences in relative abundance, but these variations are not statistically significant. This result suggests that DSS induction and *Bifidobacteriaceae* administration did not directly alter the overall abundance of this family in the gut (Fig. 6H). However, changes at co-abundance groups (CAGs) level remain possible and warrant further investigation in future studies. *Bacteroidaceae* is significantly elevated in DSS group compared with Control, while MCR-BH significantly reduced its abundance relative to both DSS and free BH (Fig. 6I). An increased Bacteroidaceae level is commonly associated with colitis-associated dysbiosis and can reflect an overrepresentation of inflammation-adapted taxa, suggesting a potential contribution to mucosal inflammation and barrier disruption.

The hierarchical clustering heatmap of the top 40 bacterial families (Fig. 6J) indicates an overall disruption of community structure after DSS induction. Samples in DSS and MCR groups show more heterogeneous and irregular abundance patterns across these dominant families, suggesting potential microbiota dysbiosis. In contrast, the MCR-BH group shows a community profile that is more similar to the healthy control group at the family level, consistent with a partial restoration of community structure.

Taken together, DSS induction drives a pronounced loss of community richness and evenness and reshapes the family-level structure toward dysbiosis configuration. Across the analysis, the MCR alone shows limited microbiota modulation because MCR tracks the DSS state in both beta-diversity and dominant-family distributions. Free BH induces only modest remodeling and does not consistently separate from the DSS-associated profile. In contrast, MCR-BH produces a coherent, multi-level restoration signal. It increases alpha diversity across richness and evenness metrics, shifts overall composition toward the control cluster in beta-diversity space, and partially re-organizes the dominant-family heatmap toward the Control group pattern. These results suggest that magnet-localized delivery strengthens the functional impact of probiotics beyond free hydrogel administration by enhancing target-site exposure and retention, thereby enabling a more robust shift toward a healthier microbial community.

## 3 Conclusion

In this study, we developed a programmable magnetic carrier robot platform for targeted probiotic delivery for the treatment of ulcerative colitis. This platform aims to address two key limitations of conventional probiotic administration: insufficient site specificity and limited intestinal retention. The MCRs are fabricated via a scalable two-step screen-printing process that integrates a programmed NdFeB/PDMS elastomer for wireless magnetic actuation. A bacteria-loaded hydrogel layer that serves as both a protective matrix and a localized-release carrier at the target region is integrated.

Functionally, the MCRs demonstrate wireless navigation and site-specific retention under real-time visualization in colon-mimicking tracks and a colon phantom, supporting their potential in clinical operation. Moreover, the platform offers practical deployment routes across anatomical scales: rectal placement is suited for large lumens such as the human colon, while in small-animal models, where robotic-arm positioning accuracy can be constrained by organ-scale dimensions, externally affixed permanent magnets provide a simple and effective means to enhance local magnetic trapping and extend retention. In further *in vivo* validation, a DSS-induced acute colitis mouse model is established, in which magnet-localized delivery of probiotic-loaded MCRs yields superior therapeutic outcomes compared with free probiotic hydrogel and material controls, demonstrating site-specific intervention and intestinal retention. Importantly, 16S rRNA analysis further demonstrated that localized delivery more effectively restored gut microbial balance than non-localized probiotic administration, with improved microbial diversity and beneficial changes in observed bacterial taxa.

In summary, MCR-BH enabled controllable navigation in *in vitro* models and regional magnetic retention in the acute mouse model, with improved clinical, histological, and microbiome outcomes relative to the tested controls. In the present study, (i) performance is validated mainly in an acute DSS colitis model with short-term follow-up, so durability and long-term safety in chronic, clinically relevant settings remain to be established, (ii) dose calibration is at a proof-of-concept stage, with therapeutic selection and per-robot loading/release requiring further standardization, and (iii) *in vivo* operation relies largely on open-loop magnetic actuation, while the feedback mechanism and closed-loop targeting remain to be developed. As noted by the current design, future work should evaluate durability, dose calibration, and long-term safety in chronic and more clinically relevant models and further explore translation-oriented delivery routes (e.g., rectal deployment in large lumens) and closed-loop targeting strategies.

## 4 Materials and methods

### 4.1 Ink preparation

Magnetic ink Preparation: To prepare the magnetic ink, we utilized a composite system consisting of magnetic particles and elastomers[37]. Magnetic particles (NdFeB, MQFPTM-16-7) were selected due to their high magnetic remanence. Before mixing, all the PrFeB particles were magnetized beyond saturation using an impulse magnetizer (IM-10-30, ASC SCIENTIFIC, USA). The elastomer matrix, PDMS (Sylgard 184, Dow Inc,US), was prepared by mixing silicone resin and curing agent at a 1:1 wt ratio. The NdFeB particles were gradually added to the matrix at a 1:1 wt ratio, and the entire formulation was blended by an overhead stirrer (NANOSTAR, IKA, Germany) in 2000 r.p.m. for 2 min to ensure homogeneity. The final mixture was degassed for 5 min in a vacuum chamber to eliminate air bubbles.

Alginate hydrogel Preparation: Sodium alginate was dissolved in sterile PBS (pH 7.4) to obtain a 10.0 wt % solution and stirred until fully hydrated overnight. The alginate solution was then degassed to remove trapped air and stored at 4 °C for subsequent use. A CaCl_2_ crosslinking solution was prepared in advance (in PBS).

For the bacteria-loaded hydrogel (BH), probiotic lyophilized powder was directly added into the prepared alginate solution and mixed thoroughly to obtain a homogeneous bacteria-alginate precursor. The resulting mixture was immediately printed onto the pre-fabricated MCR substrates to form the BH layer. Crosslinking was initiated by spraying a thin layer of CaCl_2_ solution onto the hydrogel surface, allowing rapid fixation of the printed pattern via ionic gelation. After gelation, samples were gently rinsed with sterile PBS to remove excess Ca^2+^ and were kept in sterile PBS at 4 °C before use.

### 4.2 Screen-printing procedures

The magnetic carrier robots were fabricated using a layer-by-layer screen-printing technique. A piece of 120 mesh nylon net with a laser-cut screen was tense and mounted onto a 3D-printed frame (Fig. S1). Each layer of magnetic ink was deposited by sweeping a polyurethane squeegee at an angle of around 70° and a manually controlled pressure. The substrates, including elastomer films, paper, glass, and wood, were secured onto the printing stage. After printing each layer, the ink was cured at 80 °C for 10 min on a heating platform. For multi-layered devices, additional layers were printed following the same procedure.

### 4.3 Rheological test

The rheological properties of the magnetic ink were measured using a rotational rheometer (Anton Paar MCR 302). The ink was loaded onto the parallel plate fixture with a 25 mm diameter at a 1 mm gap. A shear rate sweep was performed from 0.01 s^−1^ to 1000 s^−1^ at room temperature to determine the ink's viscosity profile and shear-thinning behavior, crucial for the screen-printing process. Additionally, oscillatory tests were conducted to measure the storage modulus (*G′*) and loss modulus (*G″*) at a constant frequency of 1 Hz, providing insights into the viscoelasticity properties of the ink.

### 4.4 Morphology characterization

Scanning electron microscopy (SEM, Thermo Fisher Apreo S, USA) was used to examine the morphologies and mesoscopic structures of the printed film. The sample was spread flat and securely affixed to the test bench before SEM characterization and analyzed at an accelerating voltage of 10 kV. This characterization allowed us to observe the printing quality and dispersion of the magnetic particles in the elastomer matrix.

### 4.5 Material mechanical characterization

The tensile tests are conducted using a texture analyzer (CT3, AMETEK GmbH B.U. Brookfield). Standardized testing samples made of PDMS/NdFeB composites and alginate hydrogels are subjected to axis tensile tests as per ASTM standard tests. The loading speed for the tests is set to 1 mm/s, and the testing range is set to 25 % strain.

### 4.6 Bacteria viability analysis

#### 4.6.1 Fluorescence staining assay for bacterial viability evaluation

The viability of probiotic bacteria during the ink formulation, hydrogel crosslinking, and bacterial release processes was evaluated using live/dead fluorescence staining. Bacterial samples were collected under four conditions: (i) bacteria suspended in PBS as the control group, (ii) bacteria incorporated into alginate printing ink, (iii) bacteria encapsulated within the crosslinked alginate hydrogel, and (iv) bacteria released after hydrogel dissolution. The viability was assessed using BacLightTM bacterial viability kit (L7012, Thermo Fisher Scientific, Waltham, MA, USA)based on SYTO-9 and propidium iodide (PI) staining. After staining, bacterial suspensions were imaged using a fluorescence microscope. Representative images were acquired under identical imaging conditions for all groups. Green fluorescence (SYTO-9-positive cells) and red fluorescence (PI-positive cells) were visualized separately, and merged images were generated to assess the overall bacterial viability during the fabrication and release processes.

#### 4.6.2 Viable bacterial enumeration

The viability of probiotics during the alginate ink formulation, screen-printing process, and hydrogel formation/release process was evaluated using the plate counting method. Three types of samples were analyzed: (i) reconstituted bacterial suspension, (ii) bacteria-loaded alginate ink after screen printing, and (iii) bacteria recovered after CaCl_2_ crosslinking and sodium citrate-mediated de-crosslinking of the alginate hydrogel. For the bacterial suspension group, lyophilized probiotics were reconstituted according to the formulation. For the bacteria-loaded alginate ink group, sodium alginate was added to the bacterial suspension to prepare 10.0 wt % alginate ink, which was subsequently screen-printed to simulate the MCR fabrication process. The printed hydrogel ink was collected, washed with PBS, and resuspended. For the hydrogel group, the printed alginate ink was crosslinked with 0.1 M CaCl_2_. The formed hydrogel was then dissolved using 1.5 wt % sodium citrate solution prepared in PBS, and the released bacteria were collected, washed, and resuspended. All samples were serially diluted in PBS, and 50 μL of the 10^−5^ dilution was spread onto de Man, Rogosa and Sharpe (MRS) agar supplemented with 0.5 wt % L-cysteine hydrochloride. The plates were incubated anaerobically at 37 °C for 24 h.

### 4.7 Animal experiment

Ethics statement: All animal experiments conformed to the guidelines for the care and use of laboratory animals and were approved by the Committee of Laboratory Animal Experimentation at Southwest University of Science and Technology (Mianyang, China). Neither the laboratory mice nor the study conflicted with the ethical guidelines, religious beliefs, or legal requirements of China.

A total of 30 Kunming mice (6–8 weeks old) were initially included and acclimated for 7 days before the experiment. Five mice were randomly assigned to the untreated healthy control group, while the remaining 25 mice underwent DSS-induced colitis modeling. To induce acute colitis, mice received drinking water containing 3 wt% DSS starting on day 0. Body weight and clinical signs were monitored daily, and the disease activity index (DAI) was calculated. To limit excessive disease severity and mortality, DSS administration was discontinued individually once a mouse reached a DAI ≥ 4, which was defined as successful induction of colitis. Mice that failed to reach a DAI ≥ 4 within 7 days of DSS exposure were considered modeling failures and excluded from subsequent treatment and analysis. Of the 25 DSS-treated mice, 20 met the predefined modeling criterion and were subsequently assigned to four treatment groups: DSS, MCRs, bacteria-loaded hydrogel (BH), and MCR-BH. Together with the five untreated healthy controls, the final analysis therefore included 25 mice in five groups, with n = 5 biological replicates per group.

*In vivo* experimental verification:

Therapeutic treatments were administered by oral gavage on days 7 and 9 after the start of DSS administration. During MCR administration, a NdFeB permanent magnet was placed on the abdomen to localize the robots in the distal colon, as confirmed by X-ray scanning and CT (Fig. 5B and Fig. S14). Throughout the treatment period, body weight and clinical scores were continuously monitored. On day 11, all mice were sacrificed, the colon was harvested, and distal colon tissues were collected and fixed for histological analysis, including hematoxylin and eosin (H&E) staining (Fig. S15).

Body weight was recorded for every animal before feeding on each of the 11 study days and normalized to the day-0 baseline. DAI was scored daily for each mouse using weight loss, stool consistency, and fecal blood, with the three component scores averaged. All 25 colons were collected on day 11, and colon length was measured from calibrated images using ImageJ.

Fecal samples were collected independently from all 25 animals and immediately frozen in liquid nitrogen. The day-11 samples were used for 16S rRNA sequencing. Shannon and Chao1 indices were calculated for alpha diversity. For beta diversity, jaccard distances were calculated across all samples and visualized by principal coordinate analysis (PCoA).

### 4.8 Histological assessment

On day 11 of the experiment, the mice were sacrificed for collecting colon samples. After washing with PBS, 1 cm of the distal section of colons was fixed in 4 % paraformaldehyde fixation and then embedded in paraffin. After being cut into round slices, the thin tissue sections were stained with H&E.

### 4.9 Gut microbiota 16s sequencing assay

After different treatments, fecal pellets were collected from mice, snap-frozen in liquid nitrogen, and sent to Novogene, Inc. for gut microbiota profiling by 16S rRNA gene sequencing. The 16S V3–V4 region was amplified using barcoded primers CCTAYGGGRBGCASCAG (forward) and GGACTACNNGGGTATCTAAT (reverse). PCR was carried out with 15 μL Phusion High-Fidelity PCR Master Mix, 0.2 μM of each primer, and ≤ 10 ng template DNA, under the following cycling conditions: 98 °C for 1 min, 30 cycles of 98 °C for 10 s, 50 °C for 30 s, and 72 °C for 30 s, and a final extension at 72 °C for 5 min. PCR products were purified using magnetic bead purification, pooled at equal-density ratios, and sequencing libraries were generated with index addition. Libraries were quantified by Qubit and real-time PCR, assessed by bioanalyzer for size distribution, and sequenced on a NovaSeq 6000 or DNBSEQ-G99 platform according to the required data output.

16S rRNA gene sequencing analysis was conducted using the Novogene bioinformatics pipeline. Paired-end reads were assigned to samples by unique barcodes and trimmed to remove barcode and primer sequences. Reads were merged using FLASH (v1.2.11) to generate raw tags, followed by quality filtering using fastp (v0.23.1) to obtain clean tags. Chimera detection was performed against the SILVA reference database, and chimeric sequences were removed using vsearch (v2.16.0) to generate effective tags. Effective tags were denoised in QIIME2 using DADA2 to infer ASVs, and taxonomic annotation was performed in QIIME2 using the SILVA database. ASV absolute abundances were normalized to the sequencing depth of the sample with the fewest reads, and downstream alpha diversity and beta diversity analyses were performed based on the normalized dataset.

### 4.10 Chemical element analysis in leaching solutions

Leaching tests of MCRs were performed by incubating 1 g of the MCR samples in 10 mL of simulated intestinal fluid (pH 7 and pH = 1) for 0–72 h. The leached elements in the solutions were identified and quantified using inductively coupled plasma mass spectrometry (ICP-MS). ICP-MS analysis was conducted using an Agilent 7900 instrument. Calibration was performed using multielement standard solutions prepared by volumetric dilution. The calibration curves were established over a concentration range of 0–1000 ppb, with correlation coefficients (R^2^) greater than 0.99. During analysis, the recovery rate of the internal standard was strictly controlled within 80–120 % to ensure analytical accuracy and reliability. The concentrations of target elements in the leaching solutions were subsequently determined by ICP-MS.

### 4.11 In vitro cytocompatibility

The in vitro cytocompatibility of the NdFeB–PDMS film was evaluated using an extract-based assay with Caco-2 cells. To prepare the MCR-conditioned medium, 50 mg of the NdFeB–PDMS film was incubated in 1 mL of Dulbecco's modified Eagle medium (DMEM; gibco) at 37 °C for 24 h. Caco-2 cells were cultured in DMEM supplemented with 10 % fetal bovine serum (Sigma-Aldrich), 1× non-essential amino acids (gibco), 1× GlutaMAX (gibco), and penicillin–streptomycin (gibco) and seeded in 96-well plates. The culture medium was subsequently replaced with the NdFeB–PDMS-film-conditioned medium, and the cells were incubated at 37 °C under 5 % CO_2_ for 12, 24, or 48 h. Cells maintained in complete culture medium served as the all-live control, whereas cells treated with 70 % ethanol for 30 min before staining served as the all-dead control. Cell viability was evaluated using calcein-AM and propidium iodide (PI) to identify viable and dead cells, respectively. Fluorescence images were acquired using a confocal laser-scanning microscope (Leica), with nominal excitation/emission wavelengths of approximately 495/515 nm for calcein-AM and 535/617 nm for PI.

## CRediT authorship contribution statement

**Zhuoyue Wang:** Writing – original draft, Visualization, Validation, Methodology, Investigation, Funding acquisition, Formal analysis, Data curation, Conceptualization. **Li Ding:** Validation, Resources, Methodology. **Dalin Zhang:** Methodology. **Sarthak Misra:** Writing – review & editing, Visualization, Validation, Supervision, Project administration, Investigation, Funding acquisition, Conceptualization. **Venkatasubramanian Kalpathy Venkiteswaran:** Writing – review & editing, Visualization, Validation, Supervision, Methodology, Investigation, Conceptualization.

## Declaration of competing interest

The authors declare that they have no known competing financial interests or personal relationships that could have appeared to influence the work reported in this paper.

## Data Availability

Data will be made available on request.
