# Supplementary material for: Biohybrid magnetic carrier robots for targeted bacteria release in ulcerative colitis
Source: Mater Today Bio. 2026 Sep 16;41:103671. doi: 10.1016/j.mtbio.2026.103671 (PMC13598171; doi:10.1016/j.mtbio.2026.103671)
Supplement: Supplementary file 1 [file mmc1.docx]

Supplementary Materials for

**Biohybrid Magnetic Carrier Robots for Targeted Bacteria Release in Ulcerative Colitis**

Zhuoyue Wang*, Li Ding*, Dalin Zhang, Sarthak Misra, Venkatasubramanian Kalpathy Venkiteswaran*

* Corresponding author.

Email: [z.wang01@umcg.nl](mailto:z.wang01@umcg.nl); dingli@swust.edu.cn; v.kalpathyvenkiteswaran@utwente.nl

The Supplementary Materials include:

Supplementary Figures:

Fig. S1. Schematic illustration of the screen-printing setup for MCR fabrication.

Fig. S2. Rheological characterization of NdFeB/PDMS composite inks.

Fig. S3. Dimensional reproducibility of screen-printed magnetic carrier robots.

Fig. S4. SEM-EDS elemental mapping of the NdFeB/PDMS magnetic layer.

Fig. S5. Structural stability and magnetic responsiveness of MCRs after prolonged exposure to pH-adjusted gastrointestinal test media.

Fig. S6. Bright-field morphology of probiotic bacteria during hydrogel formulation and release.

Fig. S7. Viable probiotic recovery during hydrogel-ink preparation, screen printing, and release.

Fig. S8. Design schematic of a molded silicone channel with transverse ridges mimicking colonic folds.

Fig. S9. Design schematic of the 150 mm planar serpentine track.

Fig. S10. Design schematic of the 75 mm planar serpentine track.

Fig. S11. Design schematic of the 30 mm planar serpentine track.

Fig. S12. Robotic control platform for magnetically driven MCR actuation.

Fig. S13. Trajectory-tracking performance of an MCR under robotic actuation.

Fig. S14. In vivo radiographic and CT verification of regional MCR localization.

Fig. S15. Histological evaluation of colonic tissues by H&E staining.

Fig. S16. Time- and pH-dependent release of Fe and Nd from magnetic carrier robots measured by ICP-MS.

Fig. S17. Cytocompatibility of MCR-conditioned medium toward Caco-2 cells.

Fig. S18. Comparison of beta-diversity among treatment groups based on Jaccard distance.

**Supplementary Movie:** Controllable Locomotion of Magnetic Carrier Robots

**Fig. S1. Schematic illustration of the screen-printing setup for MCR fabrication.**
Exploded-view diagram of the screen-printing assembly, including the squeegee, frame, mesh, fixing frame, patterned screen mold, and substrate. The configuration enables layer-by-layer deposition of functional inks to fabricate magnetic carrier robots (MCRs) with defined geometries.

**Fig. S2. Rheological characterization of NdFeB/PDMS composite inks.**

(a) Dynamic strain sweep at 1 Hz showing storage modulus (G′) and loss modulus (G″) as a function of shear strain for different NdFeB/PDMS ratios and pure PDMS. (b) Steady-shear viscosity as a function of shear rate, demonstrating shear-thinning behavior and particle-loading-dependent viscosity enhancement.

The rheological properties of the NdFeB/PDMS composite inks were systematically investigated to evaluate their printability and structural stability. Fig. (a) presents the dynamic strain sweep at a fixed frequency of 1 Hz, where the storage modulus (G′) and loss modulus (G″) were recorded as a function of shear strain. For all particle-loaded formulations, G′ exceeds G″ within the low-strain region, indicating a predominantly elastic, solid-like behavior arising from particle–polymer interactions and the formation of a percolated microstructure. Increasing the NdFeB loading significantly enhances both G′ and G″, reflecting reinforcement of the PDMS matrix by magnetic particles. The 2:1 formulation exhibits the highest moduli, suggesting stronger particle network formation. As shear strain increases, all composite inks exhibit a gradual decrease in G′, accompanied by the crossover of G′ and G″ at higher strains, indicating of structural breakdown and transition to viscous-dominated behavior. In contrast, pure PDMS shows substantially lower moduli and a more liquid-like response across the entire strain range.

The steady-shear viscosity curves in Fig. (b) further demonstrates the shear-thinning characteristics of the composite inks. All NdFeB/PDMS mixtures display pronounced viscosity reduction with increasing shear rate, which is favorable for screen-printing processes. Higher particle content leads to increased zero-shear viscosity and stronger shear-thinning behavior, attributed to particle-particle interactions and hydrodynamic resistance. Notably, the 1:1 ratio provides a balanced rheological profile, combining sufficient viscosity for pattern retention with manageable flow under applied shear. In contrast, pure PDMS exhibits the lowest viscosity and minimal shear-thinning effect, which may limit its shape fidelity after printing.

Overall, these results confirm that NdFeB incorporation effectively tailors the viscoelastic and flow properties of PDMS inks, enabling tunable printability and structural stability for multi-layer screen-printing of magnetic soft robots.

Fig. S3. Dimensional reproducibility of screen-printed magnetic carrier robots. Length and width were measured by OpenCV-based image analysis for 1,280 cross-shaped MCRs from five independently printed batches (256 MCRs per batch; total printed area, 14 cm^2^). The inset defines the length and width measurements. Violin plots show the distributions; orange boxes indicate the interquartile range, white circles indicate the median, and whiskers extend to 1.5 times the interquartile range.

To assess fabrication reproducibility, MCR dimensions from five independent printing batches were quantified using an automated image-analysis workflow. The median length and width were 1.24 and 1.20 mm, respectively, and both distributions were narrowly centered near the nominal 1.2 mm design dimension. More than 95 % of the printed MCRs showed length and width deviations of no more than 15 % of the median, supporting consistent dimensional control across batches and the reproducibility of the screen-printing process.

**Fig. S4. EDS elemental analysis of the MCR material.**

Top: SEM image of the MCR surface. Bottom: corresponding elemental mapping of Si (pink), Fe (red), Nd (green), and B (yellow), showing homogeneous distribution of NdFeB magnetic particles within the matrix. Scale bars: 300 μm.

The EDS elemental mapping confirms the compositional uniformity of the MCR material. The SEM image shows a continuous polymer matrix with dispersed microparticles. Elemental maps of Si, Fe, Nd, and B reveal homogeneous distribution across the scanned area without obvious aggregation. The co-localization of Fe and Nd signals indicates the presence of NdFeB magnetic particles, while Si is uniformly distributed, corresponding to the silica coating or matrix components. These results verify successful incorporation and dispersion of magnetic particles within the MCR matrix.

**Fig. S5. Stability and magnetic responsiveness of MCRs after prolonged exposure to SGF.**

(a) Optical image of MCR treated in SGF (pH=7) for 7 days, showing preserved structural integrity.

(b) Mechanical stretching test of MCR treated in SGF (pH=7), demonstrating retained elasticity and tensile robustness.

(c) Magnetic actuation of MCR treated in SGF (pH=7) under 40 mT field, confirming sustained deformability.

(d) Optical image of MCR treated in SGF (pH=1) for 7 days, maintaining intact morphology.

(e) Mechanical stretching of MCR treated in SGF (pH=1), showing preserved flexibility and structural stability.

(f) Magnetic deformation of MCR treated in SGF (pH=1) under 40 mT field, indicating retained magneto-mechanical functionality.

The structural integrity and magnetic functionality of the magnetic carrier robots (MCRs) were evaluated after long-term exposure to Simulated Gastric Fluid (SGF) without pepsin under different pH conditions. As shown in Fig. a–c, following 7 days of static exposure to SGF at pH=7, approximating an intestinal-like environment, the MCRs preserved their macroscopic architecture without visible damage or degradation (Fig. S4a). The material also retained high elasticity and mechanical strength, as evidenced by its ability to sustain stretching without cracking or permanent deformation (Fig. S4b). Importantly, the MCRs remained magnetically responsive; under an external magnetic field of B=40 mT, they exhibited clear, controllable deformation (Fig. S4c), confirming the function stability after prolonged intestinal retention.

Similarly, under a simulated gastric environment in SGF at pH=1, the MCRs maintained comparable durability. After 7 days of exposure, the structures remained intact with no apparent swelling, fragmentation, or degradation (Fig. S4d). Mechanical compliance was preserved (Fig. S4e), and magnetic actuation was still readily achieved under B=40 mT (Fig. S4f).

**Fig. S6. Microscopic observation of Lactobacillus under different formulation states.**
Bright-field images showing (a) bacteria in PBS (control), (b) bacterial alginate ink, (c) bacteria encapsulated in hydrogel, and (d) bacteria after hydrogel dissolution. Rod-shaped morphology is preserved across all conditions, indicating structural compatibility of the hydrogel system.

The microscopic images qualitatively compare the morphology and spatial distribution of bacteria across different formulation and processing states. In the control group (Bacteria + PBS), abundant rod-shaped Lactobacillus and Bifidobacteria cells are clearly observed with relatively uniform dispersion, serving as the reference for cell morphology and apparent cell density.

In the bacterial alginate ink, the bacteria remain predominantly well dispersed with preserved rod-like morphology, indicating that the alginate-based ink formulation does not visibly compromise cellular integrity or induce pronounced aggregation.

For the bacterial hydrogel, Lactobacillus and Bifidobacteria cells are still readily observed with intact morphology, suggesting that encapsulation within the hydrogel network is compatible with the bacteria. Notably, compared with the PBS control, the apparent bacterial density shows a slight reduction, which may reflect partial retention within the hydrogel matrix or sampling-related dilution during gel handling.

After dissolution of the bacterial hydrogel, the released bacteria maintain a morphology comparable to the control, with no obvious fragmentation or abnormal clustering. Meanwhile, the visible cell density appears modestly lower than the initial PBS control, consistent with a minor loss or incomplete recovery during the encapsulation–release process. Overall, these observations show that the bacteria-loaded hydrogel preserves Lactobacillus and Bifidobacteria morphology throughout formulation, encapsulation, and release, while exhibiting only a slight decrease in apparent cell abundance from the initial suspension to the final recovered state.

**Fig. S7. Microscopic observation of Lactobacillus under different formulation states.**
Bright-field images showing (a) bacteria in PBS (control), (b) bacterial alginate ink, (c) bacteria encapsulated in hydrogel, and (d) bacteria after hydrogel dissolution. Rod-shaped morphology is preserved across all conditions, indicating structural compatibility of the hydrogel system.

Here, a quantitative spread-plate assay with three independently prepared samples per condition. We analyzed (i) the reconstituted bacterial suspension, (ii) the bacteria-loaded alginate ink after screen printing, and (iii) the bacteria recovered after CaCl_2_ crosslinking and sodium-citrate de-crosslinking.

The samples were serially diluted in PBS, and 50 μL of the 10^-5^ dilution was plated on de Man, Rogosa and Sharpe (MRS) agar supplemented with 0.5% (w/v) L-cysteine hydrochloride. Plates were incubated anaerobically at 37 °C for 24 h, and the viability (CFU/mL) was calculated as N/(V × d), where N is the colony count, V is the plated volume, and d is the dilution factor.

The measured viable bacterial concentrations were 8.3 × 10^8^ CFU/mL in the reconstituted bacterial suspension, 1.4 × 10^8^ CFU/mL in the bacteria-loaded hydrogel ink after screen printing, and 5.2 × 10^7^ CFU/mL after gelation and release. The bacterial suspension was prepared at a nominal concentration of 2.0 × 10^9^ CFU/mL; therefore, the experimentally measured viable count represented 41.5% of the nominal value. Relative to the measured viable count of the bacterial suspension, the bacteria-loaded hydrogel ink and released bacterial suspension retained 16.9% and 6.3% viable-cell recovery, respectively. Although viable bacterial counts decreased during fabrication and gelation, the released suspension still contained 5.2 × 10^7^ CFU/mL, confirming that a substantial viable probiotic population was preserved throughout the preparation and release processes.

**Fig. S8. Design schematic of a molded silicone channel with transverse ridges mimicking colonic folds.**

Technical drawing showing top, side, cross-sectional, and isometric views of a ridged silicone channel. Periodic transverse folds are incorporated to emulate colonic haustra, enabling controlled evaluation of MCR locomotion and navigation in a biomimetic environment.

**Fig. S9. Design schematic of the large-scale planar serpentine track.**

Technical drawing of the serpentine track geometry at 150 mm × 150 mm footprint with 10 mm thickness. Top, side, and isometric views are shown, with curvature radii and channel dimensions specified to define the enlarged navigation path for MCR motion.

**Fig. S10. Design schematic of the enlarged planar serpentine track.**

Technical drawing of the scaled serpentine track geometry, including top view, side view, and isometric representation. Key dimensions (75 mm × 75 mm footprint, 5 mm thickness) and curvature radii are specified to define channel width and turning geometry for controlled MCR navigation.

**Fig. S11. Design schematic of the planar serpentine track.**

Technical drawing of the serpentine track geometry, including top view, side view, and isometric representation. Key dimensions (30 mm × 30 mm footprint, 5 mm thickness) and curvature radii are indicated to define the channel width and turning geometry for controlled MCR navigation.

**Fig. S12. Robotic control platform for magnetically driven MCR actuation.**
Photograph of the experimental setup integrating a 6-axis robotic arm, a servo-driven NdFeB disk magnet, and a planar test surface. The robotic arm enables programmable magnet motion to generate controlled magnetic fields for precise, continuous actuation and trajectory control of Magnetic Carrier Robots (MCRs).

**Fig. 13. Trajectory tracking performance of the MCR under robotic actuation.**
The red curve represents the recorded motion path, while the dashed square indicates the programmed reference trajectory. The MCR closely follows the designed square path with minor deviations at edges and corners.

The MCR trajectory demonstrates successful tracking of the pre-designed square path under robotic arm actuation. The recorded path closely follows the reference dashed square, with minor deviations along the edges and slight rounding at the corners. These discrepancies are likely attributed to magnetic response delay, frictional interaction with the substrate, and system inertia, while overall path fidelity remains high.

**Fig. S14. In vivo radiographic and CT verification of magnetic localization of MCRs.**

(a) X-ray image on Day 7 before external magnetic actuation, showing the MCR after passive transit through the gastrointestinal tract and captured in the stomach.

(b) X-ray image on Day 9 under external magnetic guidance, demonstrating successful relocation and retention of the MCR within the intestinal region. The external permanent magnet used for localization is visible beneath the animal.

(c) Three-dimensional reconstructed CT image (Day 9) confirming the spatial localization of the MCR within the intestine, providing depth-resolved validation of magnetic positioning and in vivo retention.

**Fig. S15. Histological evaluation of colonic tissues by H&E staining.**

Representative hematoxylin and eosin (H&E)–stained cross-sections of mouse colon from Control, DSS, MCR, BH, and MCR+BH groups.

Histological examination showed that the Control group displayed an intact colonic architecture with no apparent abnormalities. In contrast, the DSS group exhibited pronounced tissue disruption with moderate inflammation, accompanied by extensive crypt loss and a marked reduction in goblet cells. Similar pathological features were observed in the MCR group, which presented disrupted morphology with moderate inflammation, crypt depletion, and decreased goblet cells. The BH group showed partial structural damage with mild inflammation and limited crypt loss. Notably, the MCR+BH group largely preserved normal tissue architecture and showed only mild inflammation.

**Fig. S16. Time- and pH-dependent release of Fe and Nd from magnetic carrier robots measured by ICP-MS.** MCR composite (1 g) was incubated in 10 mL of gastrointestinal test medium adjusted to pH 1 or pH 7. Fe and Nd released after 12, 24, 48, and 72 h were measured by inductively coupled plasma mass spectrometry. Values were normalized to initial MCR mass and should be expressed as grams of released element per gram of MCR composite (g/g). Lines and shaded regions show mean +/- SD (n = 3 independent samples).

ICP-MS was used to quantify the short-term release of the principal metallic components from the NdFeB/PDMS layer under acidic and near-neutral conditions. Although Fe and Nd release gradually increased with incubation time and was higher under acidic conditions (pH 1) than under near-neutral conditions (pH 7), the detected amounts of both metallic ions remained low throughout the 72-h incubation period. Fe release was consistently higher than Nd release, reflecting the relative composition of the magnetic alloy. These results indicate that the PDMS encapsulation effectively limited metal-ion exposure from the NdFeB component and supported the short-term chemical stability of the MCR platform under simulated gastrointestinal conditions. While further long-term studies are required to fully evaluate degradation behavior and systemic exposure, these findings provide preliminary evidence supporting the short-term safety of the magnetic composite for gastrointestinal applications.

Fig. S17. Cytocompatibility of MCR-conditioned medium toward Caco-2 cells. Representative live/dead fluorescence images of Caco-2 cells exposed to NdFeB/PDMS-film-conditioned medium for 12, 24, or 48 h. Conditioned medium was prepared by incubating 50 mg of NdFeB/PDMS film in 1 mL of complete DMEM at 37 degrees C for 24 h. Calcein-AM (green) and propidium iodide (PI; red) indicate viable and membrane-compromised cells, respectively. Cells in complete medium and cells treated with 70% ethanol served as all-live and all-dead staining controls. Scale bar: 100 um.

An extract-based assay was used to evaluate whether species released from the NdFeB/PDMS layer caused acute cytotoxicity to an intestinal epithelial cell model. The staining controls produced the expected predominantly green and red signals. Cells exposed to MCR-conditioned medium remained predominantly calcein-AM positive, although the PI-positive fraction increased modestly with exposure time. ImageJ-based analysis yielded approximate viabilities of 91.5%, 86.4%, and 84.3% at 12, 24, and 48 h (n=3), respectively. These findings support short-term cytocompatibility under the tested extract conditions but do not establish long-term biosafety.

**Fig. S18. Comparison of beta-diversity among treatment groups based on Jaccard distance.** Day-11 fecal microbiota samples from the Control, DSS, MCR, BH, and MCR-BH groups were analyzed by 16S rRNA gene sequencing (n = 5 independent animals per group).

Beta diversity was evaluated using Jaccard distances calculated from the normalized feature table, and the distributions of the Jaccard-based beta-diversity indices were compared among treatment groups. Boxes indicate the median and interquartile range, and the whiskers represent the minimum and maximum values. Overall differences among groups were evaluated using the Kruskal-Wallis rank-sum test, followed by Dunn’s multiple-comparisons test for pairwise post hoc comparisons. *p < 0.05, **p < 0.01; ns, not significant. Jaccard-distance-based beta-diversity analysis was performed to compare microbial community composition among the five treatment groups and to assess whether the treatments altered the overall community structure.

The Jaccard-based beta-diversity indices differed among the treatment groups. The Control group showed higher values than the DSS and MCR groups (both p < 0.05) and the BH group (p < 0.01). The MCR-BH group showed a higher value than the BH group (p < 0.01), and its distribution approached that of the Control group. Together with the PCoA pattern, these results support treatment-associated differences in microbial community composition and suggest that MCR-BH shifted the community toward a more Control-like state.
